# Fatty Acid Profile and Some Useful Biological Aspects of Borage, Calophyllum, and Prickly Pear Seed Oils: Implications for Health and Dietary Use

**DOI:** 10.3390/antiox14060661

**Published:** 2025-05-30

**Authors:** Florinda Fratianni, Francesca Coppola, Siria Tavaniello, Maria Neve Ombra, Beatrice De Giulio, Nunzio D’Agostino, Gokhan Zengin, Raffaele Coppola, Filomena Nazzaro

**Affiliations:** 1 Institute of Food Sciences, CNR-ISA, Via Roma 64, 83100 Avellino, Italy; florinda.fratianni@isa.cnr.it (F.F.); marianeve.ombra@isa.cnr.it (M.N.O.); beatrice.degiulio@isa.cnr.it (B.D.G.); coppola@unimol.it (R.C.); 2 Department of Agricultural Sciences, University of Naples Federico II, Piazza Carlo di Borbone 1, 80055 Portici, Italy; nunzio.dagostino@unina.it; 3 Department of Agricultural Environmental and Food Sciences (DiAAA), University of Molise, Via F. de Sanctis s.n.c., 86100 Campobasso, Italy; siria.tavaniello@unimol.it; 4 Department of Biology, Faculty of Science, Selcuk University, 42130 Konya, Turkey; gokhanzengin@selcuk.edu.tr

**Keywords:** seed oils, fatty acids, antioxidants, bovine serum albumin denaturation, neuroprotective effect, biofilm

## Abstract

Seed oils from *Borago officinalis* (borage), *Opuntia ficus-indica* (prickly pear), and *Calophyllum inophyllum* (calophyllum or tamanu) are rich in bioactive fatty acids and have been traditionally used in cosmetic and industrial sectors. This study explored their fatty acid composition and investigated their in vitro antioxidant, anti-arthritic, neuroprotective, and antibiofilm activities. Fatty acid profiles were determined via gas chromatography. Antioxidant activity was assessed using DPPH and ABTS radical scavenging assays. Anti-arthritic potential was measured via bovine serum albumin denaturation. Neuroprotective properties were evaluated through acetylcholinesterase, butirylcholinesterase, and tyrosinase inhibition. Antibiofilm activity against five pathogenic strains was analyzed using crystal violet and MTT assays. Correlation analysis was used to associate fatty acid composition with bioactivity. Prickly pear oil exhibited the highest PUFA content (65.1%), mainly linoleic acid. Calophyllum oil was richer in saturated and monounsaturated fatty acids. All oils showed significant radical scavenging ability, with calophyllum oil showing the lowest DPPH IC_50_ and borage oil, the highest ABTS activity. Borage and prickly pear oils demonstrated strong anti-arthritic potential. Calophyllum oil showed the most potent AChE inhibition. All oils showed tyrosinase inhibition; however, calophyllum did not show BChE inhibitory activity. Antibiofilm activity was species- and dose-dependent, with *Staphylococcus aureus*, *Escherichia coli*, and *Acinetobacter baumannii* being most affected. Thus, the tested oils exhibited multiple biological activities, influenced by their fatty acid composition. The in vitro antioxidant, anti-arthritic, neuroprotective, and antimicrobial properties support their potential use as functional food ingredients or nutraceuticals, especially for aging-related health concerns. Further in vivo and clinical studies are needed to confirm their efficacy.

## 1. Introduction

Health and well-being have become a foundational element in promoting individual quality of life and broader economic productivity. Consumer interest in unconventional seed oils with potential health-protective properties has grown considerably in recent years. Among these, borage (*Borago officinalis*), calophyllum or tamanu (*Calophyllum inophyllum*), and prickly pear (*Opuntia ficus-indica*) seed oils have attracted increasing attention not only for their cosmetic uses but also for their nutritional and functional health benefits. Borage seed oil is notably rich in gamma-linolenic acid (GLA), an omega-6 fatty acid typically comprising 20–26% of the oil. GLA is recognized for its potent anti-inflammatory properties and has been linked to potential therapeutic effects in conditions such as rheumatoid arthritis, atopic dermatitis, and eczema. Moreover, borage oil may contribute to cardiovascular health by lowering triglyceride levels, improving HDL cholesterol, and reducing blood pressure. In addition to GLA, borage oil contains significant levels of linoleic, oleic, palmitic, stearic, erucic, and nervonic acids. It is also characterized by a high concentration of δ-tocopherol (1320 mg kg^−1^), a powerful natural antioxidant, phytosterols, and other tocopherols. Calophyllum oil is rich in essential fatty acids, primarily oleic and linoleic acids, as well as palmitic and stearic acids. It possesses anti-inflammatory, antimicrobial, and antioxidant properties and has long been used in traditional medicine for treating various skin ailments such as acne, eczema, and wounds. Importantly, calophyllum oil contains calophyllolide, a compound with anti-inflammatory and wound-healing activity, and coumarins and xanthones, which confer antimicrobial effects. Prickly pear seed oil is highly regarded in the skincare industry due to its rich content of essential fatty acids (notably linoleic, oleic, and palmitic acids), vitamin E, phytosterols, and polyphenols. These components support its antioxidant, anti-inflammatory, and hydrating functions. The oil is also associated with increased collagen production, improved skin elasticity, and reduced under-eye circles. Regarding neuroprotective potential, the current scientific literature offers limited but promising evidence regarding the effects of these oils. For instance, a study conducted in 2013 demonstrated that borage seed oil and its principal active component, GLA, extended the health span of *Drosophila melanogaster* models, suggesting protective effects against oxidative stress and genotoxicity, although the study did not directly investigate neurodegenerative diseases [1]. As for calophyllum oil, no direct studies address its effects on neurodegenerative diseases. However, its antioxidant and anti-inflammatory properties suggest a potential neuroprotective role. In 2022, Tahri-Joutey et al. investigated the anti-protective effects of prickly pear seed oil in a rat model of acute inflammation. The findings revealed a significant reduction in inflammatory markers and enhanced antioxidant enzyme activity, indicating the oil’s potential to modulate oxidative stress and inflammation—key processes in neurodegenerative disorders [2]. From a microbiological perspective, while the antimicrobial properties of borage and calophyllum oils are not extensively documented, some studies hint at their therapeutic potential. For instance, the presence of bioactive compounds in these oils may support skin health and reduce susceptibility to infection [3,4]. Calophyllum oil has also been investigated for its antiviral properties, particularly against HIV [5]. Prickly pear (*Opuntia ficus-indica*) seed oil has demonstrated a broad spectrum of biological activities, including antiviral and antibacterial properties. Spino et al. (1998) [5] first highlighted the antiviral potential of certain lipophilic C-nucleoside analogs derived from natural oils, including compounds present in calophyllum and prickly pear seed oil. More recently, Alqurashi et al. (2022) provided a comprehensive phytochemical characterization of the oil, demonstrating its significant antimicrobial, antiviral, antifungal, and anticancer activities [6]. In addition, Koubaa et al. (2017) reported that prickly pear seed oil effectively inhibited the growth of several Gram-positive bacterial strains such as *Bacillus subtilis*, *Enterococcus faecalis*, and *Bacillus thuringiensis*, as well as Gram-negative species including *Klebsiella pneumoniae*, *Escherichia coli*, *Salmonella typhimurium*, and *Enterobacter* spp., thereby confirming its broad-spectrum antimicrobial potential [7]. Therefore, these seed oils may possess notable bioactive properties with promising neuroprotective and antimicrobial potential. Nonetheless, further targeted investigations are required to comprehensively elucidate and validate their health-promoting effects. In this context, the present study aimed to characterize the chemical composition—specifically, the fatty acid profile—and to assess selected biological activities of three commercially available seed oils: borage (*Borago officinalis*), calophyllum (*Calophyllum inophyllum*), and prickly pear (*Opuntia ficus-indica*). In particular, we evaluated the in vitro inhibitory activity of these oils against cholinesterase and tyrosinase, key enzymes implicated in the pathogenesis of Alzheimer’s and Parkinson’s diseases. Additionally, we investigated their anti-arthritic potential by assessing their ability to protect a model protein, bovine serum albumin, from degradation. Finally, we examined the capacity of the oils to inhibit biofilm formation by five pathogenic bacterial strains and to impair the metabolic activity of bacterial sessile cells.

## 2. Materials and Methods

### 2.1. Oils

Three commercial seed oils of prickly pear (*Opuntia ficus-indica*), borage (*Borago officinalis*), and calophyllum (*Calophyllum inophyllum*) were bought from a local market. As specified by the manufacturers, the seeds were cold pressed, avoiding solvents. Samples were stored at 20 °C, avoiding exposure to light until the analysis.

### 2.2. Fatty Acid Analysis

The fatty acid (FA) compositions of oil samples were analyzed by GC–FID after transesterification. Fatty acid methyl esters were prepared in the presence of 2 N potassium hydroxide in methanol [8] and analyzed using a GC Trace 1600 gas chromatograph (ThermoQuest EC Instruments, Milan, Italy) equipped with a flame ionization detector (260 °C) and a fused silica capillary column (Restek Rt-2560) 100 m × 0.25 mm × 0.20 μm film thickness. Helium was used as the carrier gas. The column temperature was held at 100 °C for 5 min, then increased by 4 °C/min to 240 °C and held for 20 min. Individual fatty acid peaks were identified by comparing retention times with those of known standard fatty acid mixtures (37 Component FAME MIX Supelco, Bellofonte, PA, USA) run under the same operating conditions. Data were presented as a means of triplicate samples for each oil type, along with the standard deviation (SD). Results were expressed as a percentage of the total identified FA.

### 2.3. Antioxidant Activity Assessment

To assess the antioxidant activity of the oils, we followed the method described by Fratianni et al. [9]. The oils were combined with acetone (Sigma, Milan, Italy) in a 1:1 volume ratio. After one hour at room temperature, a 1% methanol-HCl solution (1:2, *v*/*v*) was added to each mixture. The samples were then incubated for another hour at room temperature and centrifuged at 13,000 rpm for 5 min. The supernatant was collected, and the residue was re-extracted using the same procedure. The combined supernatants were used for further analysis.

#### 2.3.1. DPPH Free Radical Scavenging Assay

The radical scavenging capacity of the seed oils was evaluated using the diphenyl-1-picrylhydrazyl (DPPH) assay in microplate format [10]. Samples were diluted 1:1 (*v*/*v*) in dimethyl sulfoxide (DMSO) and mixed with 303 μL of a methanolic DPPH solution (153 mM). Absorbance was measured at 517 nm using a UV-VIS spectrophotometer (Cary Varian, Milan, Italy). The absorbance of the DPPH solution without sample served as a control. The IC_50_ value (μg), indicating the amount of sample required to inhibit 50% of the DPPH activity in 1 mL, was calculated. All tests were run in triplicate, and results are expressed as mean ± standard deviation (SD).

#### 2.3.2. 2,2′- Azino-bis (3-ethylbenzothiazoline-6-sulfonic Acid) (ABTS) Test

The antioxidant potential of the honey was evaluated using the 2,2′-azino-bis(3-ethylbenzothiazoline-6-sulfonic acid) (ABTS) assay [11]. A 2.5 mM methanolic solution of Trolox^®^ (prepared fresh daily, Sigma-Aldrich Italia, Milano, Italy) served as the reference antioxidant standard. ABTS and potassium persulfate were each dissolved in distilled water to obtain final concentrations of 7 mM and 2.45 mM, respectively. The mixture was incubated in the dark at ambient temperature to generate the ABTS radical cation (ABTS+). This radical solution was subsequently diluted with deionized water to achieve an absorbance of 1.00 at 734 nm. Test samples (final concentrations ranging from 0.0001 to 0.0100 mg/mL) or Trolox^®^ standards (0–20 mM) were then added to the diluted ABTS+ solution, and absorbance readings were taken after 6 min of incubation. Results were expressed as the mean ± standard deviation of triplicate measurements, reported in terms of Trolox^®^ equivalent antioxidant capacity (TEAC, μmoL).

### 2.4. Anti-Arthritic Activity

The anti-arthritic effect was determined in vitro using the bovine serum albumin (BSA) denaturation assay [12,13,14]. The reaction mixture (5 mL) included 0.2 mL of a 0.5% (*w*/*v*) BSA stock solution (96% purity, Sigma, Milan, Italy) in 0.05 M Tris–phosphate buffered saline (pH 6.5), 2.8 mL of buffer, and 2 mL of sample at different concentrations. A sample containing BSA in methanol was used as a control. After heating the mixtures at 72 °C and cooling them to room temperature, absorbance was measured at 660 nm. The IC_50_ value, representing the concentration needed to inhibit 50% of BSA denaturation. Diclofenac sodium (IC_50_ = 5.5 μg) was used as a positive control. All experiments were performed in triplicate, and the results are expressed as mean values ± SD.

### 2.5. Cholinesterase Inhibition Assays

The inhibitory activities of acetylcholinesterase (AChE) and butyrylcholinesterase (BChE) were evaluated in vitro using the spectrophotometric method of Ellman et al. [15]. All assays were performed in triplicate using 96-well plates. For AChE inhibition, acetylcholine (ACh) was used as the substrate. The reaction mixture included 550 μL of 0.1 M sodium phosphate buffer (pH 8.0), 50 μL of the sample, and 5–20 ng of AChE (from *Electrophorus electricus*, 1000 U/mg). Samples and the positive control (galantamine 10 μM, Sigma-Aldrich, Milan, Italy) were dissolved in 10% DMSO. After 15 min of incubation at room temperature, DTNB (10 μL) and ACh (10 μL) were added. The release of the yellow 5-thio-2-nitrobenzoate anion was measured at 412 nm over 10 min.

For BChE inhibition, butyryl thiocholine (BCh) served as the substrate. The mixture contained 550 μL of 0.1 M potassium phosphate buffer (pH 7.0), 50 μL of sample, and 10–50 ng of BChE (from equine serum, ≥10 U/mg). After 15 min at room temperature, DTNB and BCh (10 μL each) were added, and absorbance at 412 nm was recorded after 10 min. IC_50_ values, indicating the sample concentration required to inhibit 50% of enzyme activity, were calculated. All data were expressed as mean ± SD from triplicate tests.

### 2.6. Tyrosinase Inhibition Assay

Tyrosinase inhibitory activity was assessed in vitro following the procedure by Khatib et al. [16], with minor adjustments. Samples were first diluted in DMSO. In a 96-well plate, 70 μL of phosphate buffer (pH 6.8), tyrosinase enzyme (10 U mL^−1^), and the test sample were added and incubated at 37 °C for 5 min. Then, 0.5 mM L-DOPA was introduced, and after 10 min at 37 °C, absorbance was measured at either 492 or 475 nm. Kojic acid served as the positive control, and phosphate buffer as the blank. The IC_50_ was determined as the sample concentration needed to inhibit 50% of tyrosinase activity, calculated via interpolation of the dose–response curve. Tests were carried out in triplicate, with results presented as mean ± SD.

### 2.7. Antibacterial Properties of the Oils

#### 2.7.1. Test Microorganisms and Culture Conditions

The antibacterial potential of the oils was assessed using both Gram-negative bacteria (*Acinetobacter baumannii* ATCC 19606, *Pseudomonas aeruginosa* DSM 50071, and *Escherichia coli* DSM 8579) and Gram-positive bacteria (*Listeria monocytogenes* ATCC 7644 and *Staphylococcus aureus* subsp. *aureus* ATCC 25923), provided by the Leibniz Institute DSMZ, Braunschweig Science Campus Braunschweig-Süd, GERMANY. Bacterial cultures were incubated in Luria Broth for 18 h at 37 °C (35 °C for *A. baumannii*) under constant agitation at 80 rpm (Corning LSE, Pisa, Italy) prior to testing.

#### 2.7.2. Determination of Minimal Inhibitory Concentration (MIC)

The minimum inhibitory concentration (MIC) of each oil was determined using the resazurin-based microtiter plate assay, as described by [17]. Assays were conducted in triplicate in 96-well plates, incubated for 24 h at 37 °C (or 35 °C for *A. baumannii*). MIC values were determined by observing changes in color indicative of bacterial growth inhibition.

#### 2.7.3. Inhibition of Biofilm Formation

The ability of the oils to prevent initial bacterial adhesion was assessed following the protocol by Coppola et al. [18], using flat-bottomed 96-well microtiter plates. Overnight cultures were diluted to 0.5 McFarland standard (1.5 × 10⁷ cells/mL) using fresh Luria–Bertani broth. Each well received 10 μL of bacterial suspension, 10 or 20 μg/mL of each oil sample, and Luria–Bertani broth to reach a final volume of 250 μL. Plates were sealed with parafilm to prevent evaporation and incubated for 48 h at 37 °C (35 °C for *A. baumannii*). Non-adherent cells were removed, and wells were washed twice with sterile phosphate-buffered saline (PBS). Attached cells were fixed with 200 μL methanol (15 min), stained with 2% crystal violet (20 min), washed with PBS, and air-dried. Bound dye was solubilized with 200 μL of 20% glacial acetic acid, and absorbance was measured at 540 nm. The inhibition of adhesion was calculated relative to untreated controls (0% inhibition assumed). Assays were performed in triplicate and results reported as mean ± SD.

#### 2.7.4. Evaluation of Metabolic Activity Within Biofilms

The metabolic activity of biofilm-embedded cells was evaluated using the MTT colorimetric assay [19,20]. Oils were added either at the beginning of bacterial incubation or after 24 h. After 48 h of total incubation, wells were washed and supplemented with 150 μL PBS and 30 μL of 0.3% MTT solution (Sigma, Milan, Italy). Plates were incubated for 2 h at 37 °C (35 °C for *A. baumannii*), after which the MTT solution was discarded, and wells were rinsed twice with physiological saline. Then, 200 μL of dimethyl sulfoxide (DMSO) was added to dissolve the resulting formazan crystals. After an additional 2 h incubation, absorbance was measured at 570 nm. Each experiment was carried out in triplicate, with results expressed as mean ± SD.

### 2.8. Statistical Analysis

All data were presented as mean values ± standard deviation (SD) from triplicate experiments. Antibacterial activity data were analyzed using two-way ANOVA followed by Dunnett’s multiple comparison test, with a significance threshold of *p* < 0.05. Analyses were conducted using GraphPad Prism 6.0 (GraphPad Software, San Diego, CA, USA) and MATLAB (MathWorks, MA, USA). Correlations between antibacterial activity and the fatty acid composition of the oils were evaluated using PC software “Excel Statistics” version 365 (https://www.r-project.org/).

## 3. Results

### 3.1. Fatty Acids Composition

The fatty acid composition of oils extracted from prickly pear (*Opuntia ficus-indica*), borage (*Borago officinalis*) and calophyllum (*Calophyllum inophyllum*) seeds is shown in Table 1.

In the case of prickly pear seed oil, polyunsaturated fatty acids (PUFAs) were the most abundant, making up 65.1% of the total FA, followed by saturated fatty acids (SFAs) at 18.4% and monounsaturated fatty acids (MUFAs) at 16.6%. Among the individual FA, linoleic acid (C18:2 n6) was the predominant component, accounting for 64.4%. It was followed by oleic acid (C18:1 n9) at 15.7%, and then palmitic acid (C16:0) and stearic acid (C18:0) at 13.8% and 3.8%, respectively. The fatty acid profile observed in this oil is comparable to that reported by Ennouri et al. (2005) for two species of prickly pear from Tunisia, *Opuntia ficus-indica* and *Opuntia stricta* [8]. Both species exhibited a high level of unsaturated fatty acids (88.0–88.5%), with a high level of linoleic acid (ranging from 70.3 to 74.8%), oleic acid (12.8–16.8%), palmitic acid (7.21–9.32%), and stearic acid (3.11–3.83%). Similarly, a study by Ettalibi et al. (2021) on various Moroccan varieties of *Opuntia ficus indica* and *Opuntia ficus megacantha* found that linoleic acid was the major fatty acid, comprising 60.55 to 63.46%, followed by oleic acid at 14.09 to 17.4%, and palmitic acid at 13.03 to 13.75% [21]. According to a recent review by Al-Naqeb et al. (2021), prickly pear seed oil is characterized by a high content of unsaturated fatty acids (80–88%), which includes linoleic acid (49.3–78.8%), oleic acid (12.8–25.3%), vaccenic acid (4.3–6.3%), and linolenic acid (0.23–1.1%) [22]. The main SFA identified were palmitic acid (9.3–14.3%) and stearic acid (2.2–4.3%). Variations in linoleic acid content observed across different studies may be attributed to factors such as fruit genotype or maturity stage [21]. The high proportions of unsaturated fatty acids (USFA) detected in the prickly pear seed oils are of great interest due to their nutritional and pharmaceutical potential.

### 3.2. Antioxidant Activity of the Seed Oils

In this study, we aimed to evaluate the in vitro antioxidant potential of seed oils obtained from commercially available borage, calophyllum, and prickly pear, thereby ensuring the relevance of the findings for potential consumer applications. Antioxidant activity was assessed using two well-established assays—DPPH (2,2-diphenyl-1-picrylhydrazyl) and ABTS (2,2′-azinobis-(3-ethylbenzothiazoline-6-sulfonic acid)—each performed in triplicate to ensure reproducibility and provide a comprehensive evaluation of the oils’ radical-neutralizing efficacy. The DPPH assay is widely employed to estimate the hydrogen- or electron-donating capacity of antioxidant compounds by measuring their ability to reduce the DPPH radical [23]. However, this method presents certain limitations, particularly with compounds exhibiting high steric hindrance, which may reduce their accessibility to the radical site and impair interaction [24]. In contrast, the ABTS assay offers broader applicability by enabling the evaluation of both lipophilic and hydrophilic antioxidant species over a wide pH range [11], thereby providing complementary information on the oils’ antioxidant profiles. The experimental results are summarized in Table 2.

According to the DPPH assay, all three oils demonstrated substantial antioxidant activity, as reflected by low IC_50_ values. Borage seed oil showed an IC_50_ of no more than 12.3 µg mL^−1^, while both calophyllum and prickly pear seed oils exhibited even greater potency, with IC_50_ values below 10 µg mL^−1^. Overall, the radical scavenging efficiency of the oils was superior, in most cases, to that reported by Fratianni et al. [12] for other plant-based oils such as coffee oil. Nevertheless, their activity was less pronounced compared to extracts from broccoli, coffee, and pumpkin, as previously documented.

In the ABTS assay, borage seed oil exhibited the highest antioxidant activity among the tested samples, with a value of 8.65 mM Trolox equivalents (TE)/g. In contrast, prickly pear seed oil showed lower activity (6.13 mM TE g^−1^), with calophyllum seed oil demonstrating the lowest activity (4.96 mM TE g^−1^). These data indicate that the oils were comparable to various Prunus seed oils [9]. In our experiments, the borage seed oil exhibited lower antioxidant activity than that reported by Nogala-Kalucka et al., who observed an activity of 79.35% [25], but higher than the values reported by Cui et al., who found antioxidant activity of borage seed oils from eight Chinese provinces ranging between 123.42 and 280.06 µmol TE kg^−1^ [26]. Prickly pear seed oil exhibited stronger antioxidant activity than the corresponding oil extracted from prickly pear seeds cultivated in Mexico [27]. Its antioxidant capacity was also greater than that reported by Brahmi et al. [28], and also higher than a Moroccan prickly pear seed oil (IC_50_ = 0.96 mg) [29]. Regarding calophyllum seed oil, data in the scientific literature are scarce. However, the oil analyzed in our study exhibited markedly higher antioxidant activity than previously reported by Duy An et al., who found an IC_50_ value of 50 µg [30]. Overall, these findings support the potential of borage, calophyllum, and prickly pear seed oils as valuable sources of dietary antioxidants. Despite observed variability among different oil types and experimental conditions, the results highlight the relevance of these oils in functional food and nutraceutical applications.

### 3.3. Anti-Arthritic Activity In Vitro

Antioxidant compounds found in plants and their derivatives may contribute to protecting the human body against a wide range of metabolic and degenerative diseases [31]. Dietary intake of antioxidants is inversely correlated with morbidity and mortality associated with the accumulation of degenerative components. A typical physiological response to oxidative stress is the activation of inflammatory processes, one of which—arthritis—primarily affects the elderly population. Although not curable, arthritis can be managed with the administration of nonsteroidal anti-inflammatory drugs (NSAIDs) or corticosteroids [14]. Plants are an excellent source of molecules with anti-inflammatory and anti-arthritic properties [32,33,34]. In our study, we employed the bovine serum albumin (BSA) denaturation assay to evaluate the in vitro anti-arthritic potential of borage, calophyllum, and prickly pear seed oils, following the method described by Fratianni et al., Sakat et al., and Elisha et al. [12,13,14]. The results (shown in Table 2) were expressed as IC_50_ values. Borage and prickly pear seed oils showed a significant inhibition of albumin denaturation, with IC_50_ even of 6.05 and 6.98 μg, respectively. They were more vigorous with respect to different *Cucurbitaceae* seed oils; activity was never lower than 8 μg [12] and were significantly more efficient if compared to some kinds of *Lamiaceae* honey [35].

Borage seed oil has been the subject of several investigations about its anti-inflammatory potential. Asadi-Samani et al. reviewed the chemical profile, botanical characteristics, and biological effects of *Borago officinalis*, highlighting its noteworthy anti-inflammatory properties [36]. Additionally, Gakhar recognized the therapeutic relevance of borage oil in managing conditions such as rheumatoid arthritis and atopic dermatitis [37]. These effects are largely attributed to the presence of gamma-linolenic acid (GLA), a compound known to suppress tumor necrosis factor (TNF) activity by enhancing prostaglandin E (PGE) synthesis, which in turn elevates intracellular cyclic adenosine monophosphate (cAMP) levels. The in vitro assessment of anti-inflammatory activity through protein (albumin, BSA) denaturation assays has rarely been applied to *B. officinalis* extracts. Michalak and Szopa utilized this model to assess the anti-inflammatory, antioxidant, and protective potential of *B. officinalis* extracts on skin cells, reporting promising results [38]. However, to the best of our knowledge, no prior studies have employed this assay specifically to evaluate the anti-arthritic potential of borage seed oil. Prickly pear is not only valued for its sensory appeal but also for its rich composition of bioactive compounds, including mucilage, pectin, betalains, and fructose. These components are believed to contribute to its antidiabetic, antioxidant, antiulcer, and anti-inflammatory effects [39]. Koshak and colleagues investigated the anti-inflammatory efficacy of prickly pear seed oil extracted from plants cultivated in Saudi Arabia and confirmed its activity in two rat models of inflammation [40]. Similarly, El Hachimi et al. assessed the anti-inflammatory properties of prickly pear seed oil sourced from Moroccan cultivars. Following oral administration in Swiss female mice, the oil demonstrated a significant anti-inflammatory response, with efficacy comparable to both the untreated control and the reference anti-inflammatory drug [41].

### 3.4. Inhibitory Activity of the Seed Oils Against Cholinesterases and Tyrosinase

Neurodegenerative disorders, characterized by a gradual deterioration in specific regions of the nervous system, are becoming more widespread globally, largely due to the rising number of elderly individuals. In the progression of these conditions—such as Alzheimer’s disease (AD)—levels of acetylcholine and choline drop significantly, while the enzyme acetylcholinesterase increases dramatically, contributing to neuronal dysfunction and the subsequent decline in memory and cognitive performance. Inhibiting cholinesterase activity is considered a promising strategy for preventing and managing AD. Research has primarily concentrated on γ-aminobutyric acid (GABA), the central nervous system’s primary inhibitory neurotransmitter, and certain natural substances’ influence on its function. The lack of effective therapies for neurodegenerative diseases spurred growing interest in the neuroprotective potential of plant-based compounds, especially those abundant in foods and by-products from the agro-food industry. The health-promoting effects of the traditional Mediterranean diet were initially highlighted in the Seven Countries Study and have since been reaffirmed in subsequent research. Populations that consume diets high in fruits and vegetables tend to have lower incidences of chronic conditions, such as cardiovascular disease, atherosclerosis, various cancers, and Alzheimer’s, and enjoy longer life expectancies compared to other populations. Recently, Fratianni and colleagues explored the in vitro activity of several types of *Citrus* honey and different kinds of seed oils on acetylcholinesterase (AChE), butyrylcholinesterase (BChE), and tyrosinase [12,42]. Vegetable oils, a Mediterranean diet’s cornerstone, may also defend against neurodegenerative disorders. These oils are rich in bioactive nutrients—such as linoleic and linolenic acids, monounsaturated fats, polyphenols, vitamins, carotenoids, tocopherols, and tocotrienols—that may support neuroprotection by mitigating oxidative stress. Oils like olive, corn, and perilla have demonstrated promising memory and learning impairments improvements. Our study assessed seed oils’ in vitro neuroprotective potential from borage, calophyllum, and prickly pear, specifically targeting AChE, BChE, and tyrosinase, enzymes associated with Alzheimer’s and Parkinson’s disease. Results are indicated in Table 2.

The seed oils investigated in this study demonstrated significant inhibitory activity against acetylcholinesterase (AChE), with IC_50_ values—representing the concentration required to inhibit 50% of the enzyme’s activity—remaining below 23.54 μg, as found in borage seed oil. Among the oils tested, calophyllum seed oil showed the most potent effect (IC_50_ = 19.85 μg), followed by prickly pear seed oil (IC50 = 20.13 μg). Notably, the AChE inhibition percentage was particularly remarkable in the case of calophyllum oil, which reached as high as 95%. In contrast to the relatively consistent AChE inhibition, the butyrylcholinesterase (BChE) inhibition varied more widely. Borage seed oil, which exhibited a moderate inhibitory effect on AChE, displayed a much stronger activity against BChE, with an IC_50_ of 13.2 μg. On the other hand, prickly pear seed oil was significantly less active against BChE, showing an IC_50_ of 73.2 μg—approximately five times less effective than borage seed oil. These findings are in line with previous reports on vegetal oils. For example, Collado-González et al. observed that various extra virgin olive oils did not inhibit AChE in vitro, although they were effective inhibitors of α-glucosidase and α-amylase [43]. Conversely, Barbosa Filho et al. found that olive oil acted as an AChE inhibitor in in vivo experiments using rabbits [44]. Compared to Spanish extra virgin olive oils, which exhibited IC_50_ values not lower than 483 mg for AChE and 298 mg for BChE [45], the oils tested in the present work showed far superior inhibitory potency. These outcomes confirm the earlier findings by Fratianni et al. on the significant biological properties of unconventional seed oils derived from the Rubiaceae, Cucurbitaceae, and Brassicaceae plant families [12]. The oils exhibited strong inhibitory activity against tyrosinase, with IC_50_ values ranging from 6.41 (prickly pear seed oil) to 15.75 μg (calophyllum seed oil). Borage seed oil showed an IC_50_ against tyrosinase comparable to its BChE IC_50_ value. Since tyrosinase is also involved in neurodegenerative disease mechanisms, these findings suggest that such oils may serve as effective dietary components for the elderly, pending confirmation by in vivo studies [46]. Overall, the inhibitory effects of these seed oils on enzymes associated with neurodegenerative disorders emphasize their potential beyond cosmetic use, highlighting their role as functional ingredients in health-promoting diets. The promising bioactivity of prickly pear seed oil aligns with the growing attention of the Food and Agriculture Organization of the United Nations toward unconventional oils, due to their multifunctional applications [47]. This oil contains various phytochemicals of interest for nutraceutical applications [47,48]. Similarly, borage seed oil supports previous findings by Moliner et al., who reported its neuroprotective effects in both *Caenorhabditis elegans* and Neuro-2a cell models [49], contributing to the increasing popularity of edible flowers in functional foods. These oils also have promising applications beyond the food sector, such as calophyllum seed oil, previously reviewed for biodiesel production [50]. From a food technology perspective, seed oils have demonstrated functionality in enhancing traditional products, such as the incorporation of pumpkin seed oil into buffalo meat-based Naples-style salami, which improved both nutritional and technological properties [51]. Moreover, functional dairy products fortified with borage oil, green tea, and vitamin E have shown skin barrier enhancement, as evidenced in studies on fermented milk products [52]

### 3.5. Antimicrobial Activity

Plant-derived seed oils are increasingly recognized for their antimicrobial and antibiofilm properties, which are often attributed to their complex composition, including polyunsaturated fatty acids, tocopherols, phytosterols, and various phenolic compounds. These constituents have been shown to modulate microbial growth and biofilm development through diverse mechanisms, including the disruption of membrane integrity and interference with metabolic activity [53]. The potential ability of prickly pear, borage, and calophyllum seed oil to counteract the biofilm formation of five pathogenic bacteria was evaluated using the crystal violet assay. The results are presented in Table 3.

We performed the crystal violet test (CV) to evaluate the anti-biofilm activity of borage, calophyllum, and prickly pear seed oils. This colorimetric assay is widely employed to quantify biofilm biomass by staining the extracellular polymeric matrix surrounding sessile bacterial communities. Following staining, acetic acid (20%) was used to solubilize the bound dye. Absorbance was measured at 540 nm to estimate biofilm mass. The oils were tested at two concentrations (10 µL/mL and 20 µL/mL), selected based on preliminary minimum inhibitory concentration (MIC) data. Borage seed oil demonstrated broad-spectrum antibiofilm activity at both concentrations. At 10 µL/mL, it inhibited biofilm formation by *Acinetobacter baumannii* (18.83%), *Listeria monocytogenes* (20.47%), and *Staphylococcus aureus* (38.48%). Doubling the concentration significantly enhanced the inhibitory effect, especially against *S. aureus*, where inhibition reached 63.95%. Notably, *Escherichia coli* biofilms, initially resistant to 10 µL/mL, exhibited 17.02% inhibition at 20 µL/mL, suggesting a dose-dependent response. Calophyllum seed oil showed modest antibiofilm activity at 10 µL/mL, with partial inhibition of *S. aureus* (33.47%) and *L. monocytogenes* (15.30%), while no effect was observed against *A. baumannii*, *E. coli*, or *Pseudomonas aeruginosa*. However, at 20 µL/mL, the oil exhibited consistent inhibition across all tested strains, with peak activity against *A. baumannii* (50.34%) and *S. aureus* (56.09%). This concentration-dependent behavior aligns with previous observations of essential and fixed oils, where higher doses are required to penetrate mature biofilm structures or overcome resistance mechanisms. In contrast, prickly pear seed oil was highly effective against E. coli, even at the lower concentration (76.21% inhibition), with further improvement at 20 µL/mL (80.15%). However, it exhibited no measurable activity against *L. monocytogenes* or *P. aeruginosa* at either concentration. This suggests a selective mode of action, potentially influenced by variations in outer membrane permeability or the lipid composition of the bacterial envelope [54,55,56]. Among all strains tested, *P. aeruginosa* was the most resistant to treatment, particularly against borage and prickly pear oils. This observation reinforces the challenge of targeting *P. aeruginosa* biofilms and highlights the importance of evaluating oil activity across both Gram-positive and Gram-negative species with diverse resistance profiles [57,58]. The results of the MTT assay (Table 4), used to assess the impact of borage seed oil on sessile cell metabolism, corroborate the findings of the CV assay.

At 20 µL/mL, borage oil significantly reduced the metabolic activity of sessile cells across all tested pathogens. This metabolic inhibition is consistent with biofilm suppression and may reflect compromised membrane integrity, disrupted electron transport chains, or inhibition of key metabolic enzymes [59]. In the assay conducted with borage oil, the inhibition percentages ranged from 13.30 (against *A. baumannii*) to 54.41% (against *L. monocytogenes*). Nonetheless, it is worth highlighting the notable inhibitory effect of borage oil on the metabolic activity of *S. aureus* (19.56%), *E. coli* (with inhibition of sessile cell metabolism reaching 43.56%), and *P. aeruginosa* (showing an inhibition rate of 50.83%). When comparing these findings with the results of the crystal violet assay, it can be hypothesized that in the cases of *E. coli*, *L. monocytogenes*, and *P. aeruginosa*, the mechanism of action of borage oil may predominantly involve disruption of biochemical processes crucial for sessile cell metabolism, potentially contributing to altered bacterial virulence. Conversely, for *A. baumannii* and, particularly *S. aureus*, borage oil did not appear to target sessile metabolism as the primary mechanism; rather, the observed inhibitory effects may stem from alternative pathways, which merit further investigation to clarify the underlying mechanisms. Calophyllum oil demonstrated significant inhibitory activity against the sessile cells of *A. baumannii*, *L. monocytogenes*, and *S. aureus*, with a concentration-dependent effect particularly evident for *A. baumannii*. Notably, 63.07% inhibition of sessile metabolism at 20 µL/mL suggests that calophyllum oil may interfere directly with key energy metabolism or enzymatic systems essential for biofilm maintenance and maturation. By contrast, prickly pear seed oil, previously reported to be highly effective in preventing *E. coli* biofilm formation, did not affect the metabolic activity of its sessile cells. This discrepancy indicates that the anti-biofilm effect of prickly pear oil may be primarily associated with interference in early-stage adhesion or extracellular matrix development. It is well-established that biofilm inhibition can be achieved through diverse mechanisms, including interference with quorum sensing, suppression of extracellular matrix synthesis, or modulation of surface adhesion properties [60]. Interestingly, prickly pear seed oil effectively reduced biofilm formation and sessile cell metabolism of *A. baumannii*, with inhibition rates of 31.02% and 62.71%, respectively. This dual effect underscores the variability in bacterial species’ responses to natural compounds and emphasizes the need to explore species-specific mechanisms of action. In the case of *A. baumannii*, the inhibition of sessile metabolism likely reflects a more direct impact on cell viability and biofilm robustness, which could have significant clinical relevance. Given the escalating global concern regarding antibiotic resistance, plant-derived antimicrobials—especially those capable of targeting sessile biofilm-associated cells—represent a promising alternative or adjunct to conventional therapeutic approaches. The synergistic combination of natural compounds with antibiotics has enhanced antimicrobial efficacy and reduced the likelihood of resistance emergence [61,62]. Collectively, these data support the potential of seed oils—particularly those from borage and prickly pear—as alternative or adjunctive antibiofilm agents, capable of targeting both early and mature biofilms. Their selective, dose-dependent activity against clinically relevant pathogens warrants further investigation into their mechanisms of action and synergistic potential with conventional antibiotics. We are unable to draw comparisons with other findings reported in the literature because, to the best of our knowledge, no previous studies have investigated the antibiofilm and metabolic inhibitory activities of calophyllum seed oil. Recently, Cassien et al. [63] explored the potential use of calophyllum oil from French Polynesia after evaluating certain biological properties, including its antibacterial activity, as determined by the inhibition zone assay. Indeed, the antimicrobial activity of various species within the *Calophyllum* genus is known [64]. From this perspective, no studies have reported the antibacterial activity of borage seed oil. However, numerous studies investigated the so-called Indian borage, which, despite the name, refers to a completely different species and genus, *Plectranthus amboinicus* [65]. Our results, instead, are consistent with those reported by Nazzaro et al. regarding prickly pear seed oil [66], thereby supporting existing knowledge on the antibiofilm effectiveness of this plant. For example, Blando et al. [67] demonstrated antibiofilm activity against *S. aureus* exhibited by polyphenolic extracts of *O. ficus-indica*. Moreover, Önem et al. showed that extracts obtained from the peel of *O. ficus-indica* were effective in disrupting quorum sensing mechanisms [68].

The correlation between fatty acid composition and the evaluated biological activities yielded several noteworthy findings. Most prominently, palmitic acid exhibited a strong positive influence on antioxidant activity as measured by the DPPH assay, with a correlation coefficient of ρ = −0.98, indicating that higher levels of this fatty acid were associated with enhanced radical scavenging capacity [69]. In contrast, erucic acid and γ-linolenic acid appeared to exert an inhibitory effect on antioxidant performance in the same assay, with correlation coefficients of ρ = 0.94 and ρ = 0.90, respectively. In the ABTS assay, eicosanoic acid demonstrated the most significant contribution to antioxidant activity, showing a strong correlation (ρ = 0.94) with improved performance across the three oils tested. Regarding the anti-arthritic activity of the oils, evaluated in terms of IC_50_, stearic acid and oleic acid emerged as the most influential fatty acids, both showing strong correlations (ρ = −0.97), suggesting enhanced anti-arthritic effects with increasing concentrations of these compounds [70,71]. Palmitic acid also contributed to this activity, albeit to a lesser extent (ρ = 0.70). All other fatty acids displayed only weak associations with anti-arthritic effects, with linoleic acid standing out for its relatively stronger suppressive impact on this activity (ρ = 0.72).

The relationship between the fatty acid profiles of the three seed oils and their inhibitory effects on both biofilm formation by pathogenic microorganisms and the metabolic activity of sessile bacterial cells underscores the potential role of fatty acids as antimicrobial and anti-biofilm agents. Fatty acids can interfere with multiple aspects of bacterial physiology, including cell membrane integrity, motility, quorum sensing, and virulence factor production, which are all crucial for biofilm development and maintenance [72]. Given the differential responses of various bacterial species to natural and synthetic fatty acids, elucidating the structural and functional attributes that confer antimicrobial and antibiofilm activity is essential. Such insights may lead to developing novel therapeutic strategies to combat persistent infections caused by biofilm-forming and multidrug-resistant pathogens. Correlation analysis revealed that, in the case of *A. baumannii*, palmitic acid significantly influenced both biofilm inhibition (ρ = 0.91) and the inhibition of sessile cell metabolism (ρ = 0.92). Among the other fatty acids, only oleic acid (ρ = 0.88) showed a strong association with the oils’ inhibitory activity against the biofilm formation of this pathogen. The inhibitory activity exhibited by the oils against *E. coli* biofilm formation was primarily influenced by linoleic acid. In contrast, erucic acid (ρ = 0.89), eicosanoic acid (ρ = 0.92), and γ-linolenic acid (ρ = 0.91) were the fatty acids most strongly associated with the suppression of the metabolic activity of *E. coli* sessile cells. In the case of *L. monocytogenes*, erucic acid, linoleic acid, and α-linolenic acid appeared to exert a comparable influence on both biofilm formation (ρ = 0.87, 0.87, and 0.82, respectively) and the metabolism of sessile cells (ρ = 0.81, 0.82, and 0.81, respectively). For *P. aeruginosa*, the inhibitory effect of the seed oils on biofilm development seemed to be mainly driven by stearic acid and oleic acid (ρ = 0.91 and 0.89, respectively). In contrast, oleic acid (ρ = 0.61), and to a lesser extent erucic, eicosanoic, and γ-linolenic acids (ρ = 0.51, 0.52, and 0.51, respectively), appeared to be involved in the inhibition of the metabolic activity of *P. aeruginosa* sessile cells. The inhibitory effect of the oils against *S. aureus* biofilm formation did not appear to be significantly associated with any fatty acid. The highest correlation coefficient observed in this case was ρ = 0.67. Unlike the trends noted for the other pathogens, the correlation analysis between the quantitative fatty acid profiles and the inhibitory effects against both biofilm formation and sessile cell metabolism of *A. baumannii* and *E. coli* revealed a predominance of negative correlation values across nearly all fatty acids. The correlation analysis between the inhibitory activities of the oils and their fatty acid composition revealed that palmitic acid was the most influential compound in modulating AChE inhibition. Specifically, a strong positive correlation was observed when considering the inhibitory activity as a percentage (ρ = 0.78). A strong negative correlation was found when calculated in terms of IC_50_ values (ρ = −0.94), confirming that higher palmitic acid content was associated with enhanced AChE inhibitory potency [73]. However, palmitic acid exerted an inverse effect on BChE inhibition, displaying a strong negative correlation with the percentage inhibition (ρ = −0.94) and a negligible correlation when assessed via IC_50_ values (ρ = −0.03). In contrast, BChE inhibition appeared to be positively influenced by the presence of erucic acid and eicosanoic acid, both exhibiting a strong correlation (ρ = 0.94) with BChE inhibition expressed as a percentage, confirming at least the beneficial neuropharmacological effects of erucic acid [74]. Conversely, linoleic acid demonstrated a suppressive effect on AChE inhibition, as evidenced by its negative correlation with percentage inhibition (ρ = −0.28) and a positive correlation with IC_50_ values (ρ = 0.80), suggesting reduced effectiveness in the presence of this fatty acid. Interestingly, linoleic acid showed a strong negative correlation with tyrosinase inhibition (ρ = −0.89), indicating a potentially significant role in enhancing tyrosinase-inhibitory activity, as also demonstrated by Ando et al. [75]. Future research will be addressed to investigate the potential interactions with other food components, such as hormones, or drugs. Such interactions may impact the safety and efficacy of these oils, particularly in clinical contexts. Future studies should therefore aim to evaluate their pharmacological and metabolic efficacy. The oils used in this study came from commercial sources, to reflect products commonly used by consumers or in clinical settings. As the chemical composition of vegetable oils may vary depending on botanical origin, cultivar, geographical location, and production techniques, these factors may influence their biological activity and safety. Thus, it will be appealing to compare the behavior shown by the oils obtained from different plant varieties and if and how the extraction processes influence their chemical composition and bioactivity.

## 4. Conclusions

The biological activities exhibited by borage, calophyllum, and prickly pear seed oils—traditionally employed in non-food sectors such as cosmetics and, in the case of calophyllum oil, even as biodiesel—are increasingly gaining attention for their potential applications in the health and nutrition sectors. These oils’ significant antioxidant and antibiofilm properties suggest promising uses in food technology, particularly as natural agents for preservation and quality enhancement. Their incorporation could help extend shelf life and improve food products’ safety and organoleptic characteristics, aligning with the growing demand for clean-label, functional ingredients. Moreover, these seed oils possess notable nutraceutical features, including anti-inflammatory effects and the ability to inhibit key enzymes involved in neurodegenerative processes. These bioactivities underscore their potential inclusion—either as individual ingredients or as functional components of enriched food formulations (e.g., snacks, filled pastries, or cookies)—in diets tailored to the elderly population, a population steadily increasing worldwide. While the current findings are based primarily on in vitro studies, they are strongly supported by existing in vivo data from the international literature, confirming these oils’ biological efficacy in cellular and animal models. Future investigations, particularly clinical studies, will be essential to validate these promising results and further explore their integration into functional foods and preventive health strategies.

## Figures and Tables

**Table 1:** Mean and standard deviation of fatty acid composition of prickly pear seeds, borage (*Borago officinalis*) and *Calophyllum inophyllum* oils (% of total fatty acids) SFA: saturated fatty acids; MUFA: monounsaturated fatty acids; PUFA: polyunsaturated fatty acids.

*Items*	*Opuntia* *Ficus-indica*	*Borago officinalis*	*Calophyllum inophyllum*
C16:0	13.83 ± 1.17	10.70 ± 0.21	15.75 ± 1.04
C17:0	0.05 ± 0.00	0.06 ± 0.00	0.13 ± 0.04
C18:0	3.79 ± 0.42	5.97 ± 0.18	14.08 ± 0.17
C20:0	0.34 ± 0.02	0.37 ± 0.01	0.74 ± 0.08
C22:0	0.22 ± 0.06	0.23 ± 0.01	0.24 ± 0.03
C24:0	0.14 ± 0.02	0.10 ± 0.00	0.09 ± 0.03
SFA	18.36 ± 1.69	17.43 ± 0.42	31.03 ± 0.96
C16:1	0.68 ± 0.06	0.13 ± 0.00	0.29 ± 0.02
C18:1n9	15.71 ± 0.09	21.32 ± 0.26	38.13 ± 0.71
C20:1	0.21 ± 0.01	3.98 ± 0.10	0.23 ± 0.09
C22:1	-	2.27 ± 0.07	-
C24:1	-	1.48 ± 0.06	-
MUFA	16.59 ± 0.02	29.18 ± 0.49	38.65 ± 0.78
C18:2n6	64.43 ± 1.68	35.91 ± 0.26	29.77 ± 1.75
C18:3n6	-	17.16 ± 0.64	-
C18:3n3	0.45 ± 0.01	0.20 ± 0.01	0.25 ± 0.01
C20:2n6	-	0.14 ± 0.00	-
C20:3n3	0.18 ± 0.00	-	0.29 ± 0.21
PUFA	65.06 ± 1.68	53.40 ± 0.91	30.32 ± 1.54
PUFA n3	0.63 ± 0.01	0.20 ± 0.01	0.54 ± 0.21
PUFA n6	64.43 ± 1.68	53.21 ± 0.90	29.77 ± 1.75

**Table 2:** Biochemical characterization of borage, calophyllum, and prickly pear seed oils. Results are the average of three independent experiments (±SD). Different letters over the lines indicate statistically significant differences (*p* < 0.05) according to ANOVA followed by Dunnett’s multiple comparison test; nd: not detected.

	Borage	Calophyllum	Prickly Pear
DPPH (IC_50_, μg)	12.3 ± 1.46 ^c^	8.07 ± 0.19 ^c^	9.47 ± 0.67 ^c^
DPPH (%)	71.02 (±2.01) ^c^	79.8 (±1.96) ^c^	76.6 (±2.14) ^c^
TEAC (mM TE g^−1^)	8.65 ± 0.29 ^c^	4.96 ± 0.39 ^c^	6.13 ± 0.11 ^c^
Anti-arthritic activity (IC_50_, μg)	6.05 ± 0.25 ^c^	nd	6.98 ± 0.7 ^c^
AChE-inhibitory activity (%)	62.8 ± 1.8 ^b^	95 ± 2.3 ^c^	62.4 ± 0.13 ^b^
AChE-inhibitory activity (IC_50_, μg)	23.54 ± 1.8 ^b^	19.85 ± 2.82 ^b^	20.13 ± 0.13 ^b^
BchE-inhibitory activity (%)	71.6 ± 2.48 ^b^	nd	5.4 ± 0.67 ^a^
BchE-inhibitory activity (IC_50_, μg)	13.2 ± 1.17 ^c^	nd	73.2 ± 0.67 ^a^
Tyrosinase-inhibitory activity (DOPA, IC_50_, μg)	13.5 ± 0.67 ^c^	15.75 ± 1.67 ^c^	6.41 ± 0.23 ^c^

**Table 3:** Inhibitory activity of borage, calophyllum, and prickly pear seed oils against the biofilm formation of *A. baumannii*, *E. coli*, *L. monocytogenes*, *P. aeruginosa*, and *S. aureus*. Results were compared to the control, for which we assumed an inhibition = 0. Data are expressed in terms of percentage (SD). Different letters over the lines indicate statistically significant differences (*p* < 0.05); nd: not detected.

	Borage Seed Oil 10 μL/mL	Borage Seed Oil 20 μL/mL	Calophyllum Seed Oil 10 μL/mL	Calophyllum Seed Oil 20 μL/mL	Prickly Pear 10 μL/mL	Prickly Pear 20 μL/mL
*A. baumanii*	18.83 (±1.13) ^b^	24.93 (±2.12) ^b^	0.00 (±0.00) ^nd^	50.34 (±4.02) ^b^	19.05 (±1.13) ^b^	31.02 (±2.67) ^b^
*E. coli*	0.00 (±0.00) ^nd^	17.02 (±0.98) ^b^	0.00 (±0.00) ^nd^	34.28 (±2.77) ^b^	76.21 (±2.98) ^c^	80.15 (±2.12) ^c^
*L. monocytogenes*	20.47 (±1.98) ^b^	31.87 (±3.02) ^b^	15.30 (±0.85) ^b^	15.56 (±0.92) ^b^	0.00 (±0.00) ^nd^	0.00 (±0.00) ^nd^
*P. aeruginosa*	0.00 (±0.00)	0.00 (±0.00)	8.91 (±0.33) ^a^	39.05 (±2.15) ^b^	0.00 (±0.00) ^nd^	0.00 (±0.00) ^nd^
*S. aureus*	38.48 (±2.32) ^b^	63.95 (±2.14) ^c^	33.47 (±2.67) ^b^	56.09 (±4.44) ^c^	28.02 (±1.67) ^b^	28.42 (±1.65) ^b^

**Table 4:** Inhibitory activity of borage, calophyllum, and prickly pear seed oils against the metabolism of the sessile cells during the biofilm formation of *A. baumannii*, *E. coli*, *L. monocytogenes*, *P. aeruginosa*, and *S. aureus*. Results were compared to the control, for which we assumed an inhibition = 0. Data are expressed in terms of percentage (SD). Different letters over the lines indicate statistically significant differences (*p* < 0.05); nd: not detected.

	Borage Seed Oil 10 μL/mL	Borage Seed Oil 20 μL/mL	Calophyllum Seed Oil 10 μL/mL	Calophyllum Seed Oil 20 μL/mL	Prickly Pear 10 μL/mL	Prickly Pear 20 μL/mL
*A. baumanii*	0.00 (±0.00) ^nd^	13.30 (±0.57) ^a^	34.41 (±4.01) ^b^	63.07 (±3.45) ^c^	51.28 (±4.12) ^c^	62.71 (±2.87) ^c^
*E. coli*	26.23 (±2.04) ^b^	43.56 (±2.12) ^b^	0.00 (±0.00) ^nd^	0.00 (±0.00) ^nd^	0.00 (±0.00) ^nd^	0.00 (±0.00) ^nd^
*L. monocytogenes*	21.35 (±1.15) ^b^	54.41 (±3.33) ^c^	25.67 (±3–14) ^b^	31.86 (±2.09) ^b^	0.00 (±0.00) ^nd^	0.00 (±0.00) ^nd^
*P. aeruginosa*	0.00 (±0.00) ^nd^	50.83 (±4–04) ^b^	0.48 (±0.00) ^nd^	7.04 (±0.14) ^a^	0.00 (±0.00) ^nd^	49.52 (±3.12) ^b^
*S. aureus*	0.00 (±0.00) ^nd^	19.56 (±0.67) ^b^	11.28 (±1.09) ^a^	33.49 (±3–03) ^b^	0.00 (±0.00) ^nd^	0.00 (±0.00) ^nd^

## Data Availability

All data are shown in this paper.
